# Identification of high-risk patterns of myopia in Chinese students based on four major behavioral risk factors: a latent class analysis

**DOI:** 10.1186/s12889-023-15963-7

**Published:** 2023-07-18

**Authors:** Dan-Lin Li, Zhi-Jian Yin, Yue-Zu Li, Ya-Jie Zheng, Yu Qin, Gang Liang, Chen-Wei Pan

**Affiliations:** 1 School of Public Health, Suzhou Medical College of Soochow University, 199 Ren Ai Road, Suzhou, 215123 China; 2 Department of Ophthalmology, the First Affiliated Hospital of Dali University, Dali, China; 3 Department of Ophthalmology, the Affiliated Hospital of Yunnan University, Kunming, China; 4 Department of Ophthalmology, the Second People’s Hospital of Yunnan Province, Kunming, China

**Keywords:** Behaviors, Myopia, Ocular biometric parameter, Latent class analysis

## Abstract

**Background:**

Myopia is prevalent in children and adolescents. Understanding the effect of multiple behaviors and their latent patterns on ocular biometric parameters may help clinicians and public health practitioners understand the behavioral risk pattern of myopia from a person-centered perspective. The purpose of this study was to identify the patterns of four major behavioral risk factors associated with myopia, including time spent outdoors, digital screen time, sleep duration, and performance of Chinese eye exercises. The study also examined the relationships between these behavioral patterns and myopia as well as ocular biometric parameters in a sample of Chinese college students.

**Methods:**

This study included 2014 students from the Dali University Students Eye Health Study. The average age of the subjects was 19.0 ± 0.9 years old, ranging from 15.7 to 25.1 years old. Each participant’s refractive status was measured using an autorefractor without cycloplegia and ocular biometric parameters were measured using an IOL Master. Behavioral risk factors were collected using a pre-designed self-administered questionnaire. Latent class analysis (LCA) was performed to identify cluster patterns of various behaviors.

**Results:**

The prevalence of myopia was 91.8% in this population. The 2-class model was selected for the LCA based on goodness-of-fit evaluation metrics. Among the overall study sample, 41.1% and 58.9% were assigned into the high-risk and low-risk class, respectively. The risk of myopia [odds ratio (OR) = 2.12, 95% confidence interval (CI) = 1.52–3.14], high myopia (OR = 1.43, 95% CI = 1.14–1.78) and axial length/corneal radius (AL/CR) ratio of more than 3.0 (OR = 1.82, 95% CI = 1.22–2.72) were significantly higher in the high-risk compared with low-risk class.

**Conclusions:**

Chinese university students showed differential risks of myopia and could be subdivided into high- and low-risk clusters based on four behavioral variables.

## Introduction

Myopia is a global public health concern and its burden has been increasing rapidly worldwide in recent decades [1]. It is estimated that nearly 2.7 billion of the world’s population was myopic in 2020, of which approximately 700 million were in China [2]. Myopia is not a benign disorder and could lead to pathological changes in the retina and even cause blindness [3]. Thus, precise identification of individuals who are at high risk is crucial to guide myopia prevention and early intervention.

It has been well established that behavioral risk factors such as time spent outdoors [4], digital screening time [5] and sleep duration [6] play an important role in myopia development. Moreover, Chinese eye exercise is a mandatory measure introduced by the Chinese National Education Commission during the school years aiming at reducing the risk of myopia among Chinese school students. The frequency and quality of Chinese eye exercise have been proven to affect myopia risks among Chinese students [7, 8]. Most previous studies have assessed the individual or joint effects of these behavioral risk factors on myopia development but have neglected the cluster effects [6, 9]. Considering that these individual behavioral risk factors often do not occur individually but simultaneously and their health effects are not simply additive, it is important to understand how these behavioral risk factors co-occur or cluster [10, 11], which would help with the precise identification of high-risk behavioral patterns of myopia among school students.

Latent class analysis (LCA) is a useful method for demonstrating potential heterogeneity and efficiently identifying cluster patterns of various behaviors from a person-centered perspective, rather than a variable-centered one [12]. It treated similar individuals as one class based on a data-driven exploratory method and can be used to identify the sub-phenotypes of diseases or behaviors [13]. Although LCA analyses have proven effective in identifying the impact of high-risk behavioral patterns on health outcomes in recent years [10, 14, 15], no such studies have been conducted in the field of myopia research to date. Considering that myopia is a disorder driven by multiple behavioral risk factors, the LCA might be a promising approach that could integrate different behavioral risk factors and identify the high-risk behavioral patterns.

The objective of this study was to identify the patterns of four major behavioral risk factors associated with myopia including time spent outdoors, digital screen time, sleep duration and the performance of Chinese eye exercise using the LCA approach. In addition, we examined the relationships of the behavioral patterns with myopia and ocular biometric parameters including axial length (AL), corneal radius (CR), and anterior chamber depth (ACD) in a sample of Chinese college students.

## Methods

### Study participants

The Dali University Students Eye Health Study is a natural extension of two previous studies including the Yunnan Minority Eye Study on middle-aged to elderly adults [16] and the Mojiang Myopia Progression Study on primary and secondary school students in Yunnan Province located in southwestern China [17]. The Dali University Students Eye Health Study aims to provide university-based data on the risk factors of visual impairment and common eye disorders among university students in Yunnan Province, which would help with the understanding of prevalence of ocular problems and facilitating health service planning and medical resource allocation in relatively underdeveloped areas of China. Detailed study protocols have been described in a previous report [18]. Briefly, the sampling frame included all freshmen entering the Dali University in 2021 and the list of their names was obtained from the university admissions department. The research assistant collected the information of name, identification number, date of birth and mobile phone number of each student and sent a text message to all students in the list to invite them to participate. A subsequent telephone call with detailed study information explained by research assistants was made and the students were invited for a free eye check-up at an appointed date and time at campus if they fulfilled the eligibility criteria. Students were excluded from the study if they were older than 26 years or had eye diseases other than myopia (e.g., keratoconus, acute infection). Finally, a total of 2014 (response rate 74.7%) students participated in the study and completed both questionnaires and eye examinations. There were no differences in terms of age and sex between responders and non-responders (*P* > 0.05).

The Dali University Students Eye Health Study was conducted in accordance with the Declaration of Helsinki and ethics committee approval was obtained from the Affiliated Hospital of Yunnan University. Informed consent was obtained from each participant before enrollment.

### Eye examinations

Each participant’s refractive status was measured by a research ophthalmologist (Yue-Zu Li) using an autorefractor (KR800, Topcon) without cycloplegia. Spherical equivalent (SE) was calculated as the sum of spherical and one-half of the cylindrical. Myopia was defined as a SE less than − 0.5 diopter (D), and high myopia, as a SE less than − 6.0 D [19]. Ocular biometric parameters of the participants were measured using a non-contact optical biometry machine (IOL Master, Zeiss). AL refers to the distance from the front of cornea to the beginning of the retina, including the thickness of cornea, anterior chamber, lens, vitreous, and retina [20]. CR is the light reflectivity of the cornea [21]. ACD was measured from the anterior corneal surface and the anterior crystalline lens surface [21]. ALs, ACDs and CRs in the horizontal and vertical meridian were measured in right and left eyes. As the correlations of ALs (*r* = 0.85), CRs (*r* = 0.90), and ACDs (*r* = 0.89) between two eyes were high, only the data of the right eyes are presented. The axial length/corneal radius (AL/CR) ratio was considered an important determinant of myopia and a value greater than 3.0 was defined as a higher AL/CR ratio in this study.

### Assessment of behavioral risk factors

Behavioral risk factors including digital screen time, time spent outdoors, sleep durations and the performance of Chinese eye exercises were collected using a pre-designed self-administered questionnaire. Digital screen time was estimated by summarizing time spent on watching TV and using computers, iPads, mobile phones, or other digital products per day. Time outdoors questions included time engaged in the outdoor activities such as running, swimming, bicycle riding, attending PE lessons, exercise between classes and other outdoor activities. Night sleep duration was estimated by asking the participants the sleep and wake up time per day. The performance of Chinese eye exercise was self-evaluated by the participants with two options provided (high vs. low quality).

### Statistical analyses

The candidate behavioral risk factors were categorized into four binary indicator variables including digital screen time per day (2 h or more per day vs. less than 2 h), time spent outdoors per day (2 h or more per day vs. less than 2 h), sleep duration per night (7 h or more vs. less than 7 h) and the performance of Chinese eye exercises (high vs. low quality). The LCA is a model-based clustering approach and was conducted to analyze the four indicator variables using Mplus version 7.4 (Muthén & Muthén, Los Angeles, CA, USA). The LCA assumes that heterogeneous populations are a mixture of population and this method classifies the population by probability. Each individual belongs to a cluster with a certain probability and is ultimately assigned to the cluster with the highest posterior probability. To be specific, the LCA identifies the patterns of individuals based on the underlying potential configuration of four behavioral risk factors of myopia mentioned above. The evaluation of the model fit involves several indicators including entropy, Akaike information criterion (AIC), Bayesian information criterion (BIC), adjusted BIC, Lo-Mendell-Rubin Likelihood Ratio (LMR-LRT) and Bootstrapped Likelihood Ratio Tests (BLRT). Among them, the entropy represents the model separation clarity ranging from “0” to “1”, and being closer to “1” denotes better model separation [22]. Furthermore, lower AIC, BIC, and adjusted BIC values represent a better model fit [23]. Regarding LMR and BLRT, a *P* value of less than 0.05 indicates that the model fit was better than the model with one less classification.

Chi-square tests and student t-tests were performed using SPSS 23.0 (SPSS Inc, Chicago, IL, USA) to compare the demographic variables, prevalence of myopia and ocular biometry parameters between different clusters. Logistic regression models were used to compare the association of different behavior types with myopia, high myopia and graeter AL/CR ratio. A P value of less than 0.05 was considered to be statistically significant.

## Results

A total of 2014 students including 637 male and 1377 female students contributed to the current analysis with a mean age of 19 0.0 ± 0.9 years old, ranging from 15.7 to 25.1 years old. The prevalence of myopia and distributions of ocular biometric parameters are shown in Table 1. A total of 1848 (91.8%) students were myopic and female students had a higher prevalence of myopia (94.0% vs. 87.0%, *P* < 0.001) compared with male ones. The mean AL, CR, ACD were 24.81 ± 1.21 mm, 7.87 ± 0.32 mm and 3.62 ± 0.25 mm, respectively. In addition, male students had longer ALs, flatter CRs, higher AL/CR ratios and deeper ACDs compared with female ones (all *P* < 0.05). Participants from urban areas or had more educated parents tended to have longer ALs and higher AL/CR ratios.

**Table 1:** Differences in myopia and four ocular biometry parameters in demographic variables(*n* = 2014)

Variable	Myopia			AL(mm)		CR(mm)		AL/CR		ACD(mm)	
	No	Yes	*P*	$\bar x \pm S$	*P*	$\bar x \pm S$	*P*	$\bar x \pm S$	*P*	$\bar x \pm S$	*P*
Sex											
Male	83(13.0)	554(87.0)	**< 0.001**	25.18 ± 1.27	**< 0.001**	7.95 ± 0.31	**< 0.001**	3.17 ± 0.16	**0.03**	3.69 ± 0.25	**< 0.001**
Female	83(6.0)	1294(94.0)		24.65 ± 1.14		7.83 ± 0.31		3.15 ± 0.14		3.24 ± 0.24	
Ethnicity											
Han	120(7.9)	1391(92.1)	0.40	24.83 ± 1.20	0.49	7.87 ± 0.33	0.84	3.16 ± 0.15	0.48	3.62 ± 0.24	0.30
Minority	46(9.1)	457(90.9)		24.79 ± 1.23		7.87 ± 0.27		3.15 ± 0.14		3.61 ± 0.25	
Local resident											
Yes	119(7.7)	1420(92.3)	0.13	24.79 ± 1.21	0.05	7.87 ± 0.32	0.59	3.15 ± 0.15	0.13	3.62 ± 0.24	0.98
No	47(9.9)	428(90.1)		24.91 ± 1.21		7.87 ± 0.30		3.17 ± 0.14		3.62 ± 0.25	
Habitual residence											
Rural	112(8.5)	1209(91.5)	0.60	24.72 ± 1.20	**< 0.001**	7.85 ± 0.33	**< 0.001**	3.15 ± 0.15	**0.02**	3.61 ± 0.25	0.07
Urban	54(7.8)	639(92.2)		25.01 ± 1.21		7.90 ± 0.28		3.17 ± 0.15		3.63 ± 0.24	
Father’s education level											
< High school degree	131(8.5)	1408(91.5)	0.43	24.73 ± 1.18	**< 0.001**	7.85 ± 0.32	**< 0.001**	3.15 ± 0.15	**0.01**	3.61 ± 0.24	0.25
≥ High school degree	35(7.4)	440(92.6)		25.10 ± 1.23		7.92 ± 0.29		3.17 ± 0.15		3.62 ± 0.25	
Mother’s education level											
< High school degree	140(8.4)	1532(91.6)	0.64	24.75 ± 1.18	**< 0.001**	7.86 ± 0.32	**0.004**	3.15 ± 0.14	**< 0.001**	3.61 ± 0.24	**0.02**
≥ High school degree	26(7.6)	316(92.4)		25.19 ± 1.27		7.91 ± 0.30		3.19 ± 0.16		3.65 ± 0.25	

Bolded number represents the *P* < 0.05. Statistical methods: Chi-square test and t - test; AL, axial length; CR, corneal radius; ACD, anterior chamber depth. $\bar x \pm S$: mean ± standard deviation

Table 2 shows the indicator information of the LCA models using different number of classes. Although the entropy in the 2-class model was smaller, it was close to the good class separation criterion (0.6). The AIC, BIC and adjusted-BIC were lowest in the 2-class model. Moreover, the *P* values of LMR-LRT and BLRT were significant in the 2-class model (both *P* < 0.05). Therefore, the 2-class model was selected in the current analysis.

**Table 2:** Model fit and class descriptions of LCA models

Classes	*df*	AIC	BIC	Adjusted BIC	LMR-LRT	BLRT	Entropy	Classification probability
**2**	**9**	**8524.84**	**8575.31**	**8546.71**	**0.02**	**0.02**	**0.58**	**0.41** **0.59**
3	14	8528.63	8607.15	8562.67	0.06	0.07	0.87	0.00 0.08 0.92

Bolded row represents the selected model

*df*, degrees of freedom; AIC, Akaike information criterion; BIC, Bayesia information criterion; LMR-LRT, Lo-Mendell-Rubin Likelihood Ratio; BLRT, Bootstrapped Likelihood Ratio Tests

**Fig. 1:**
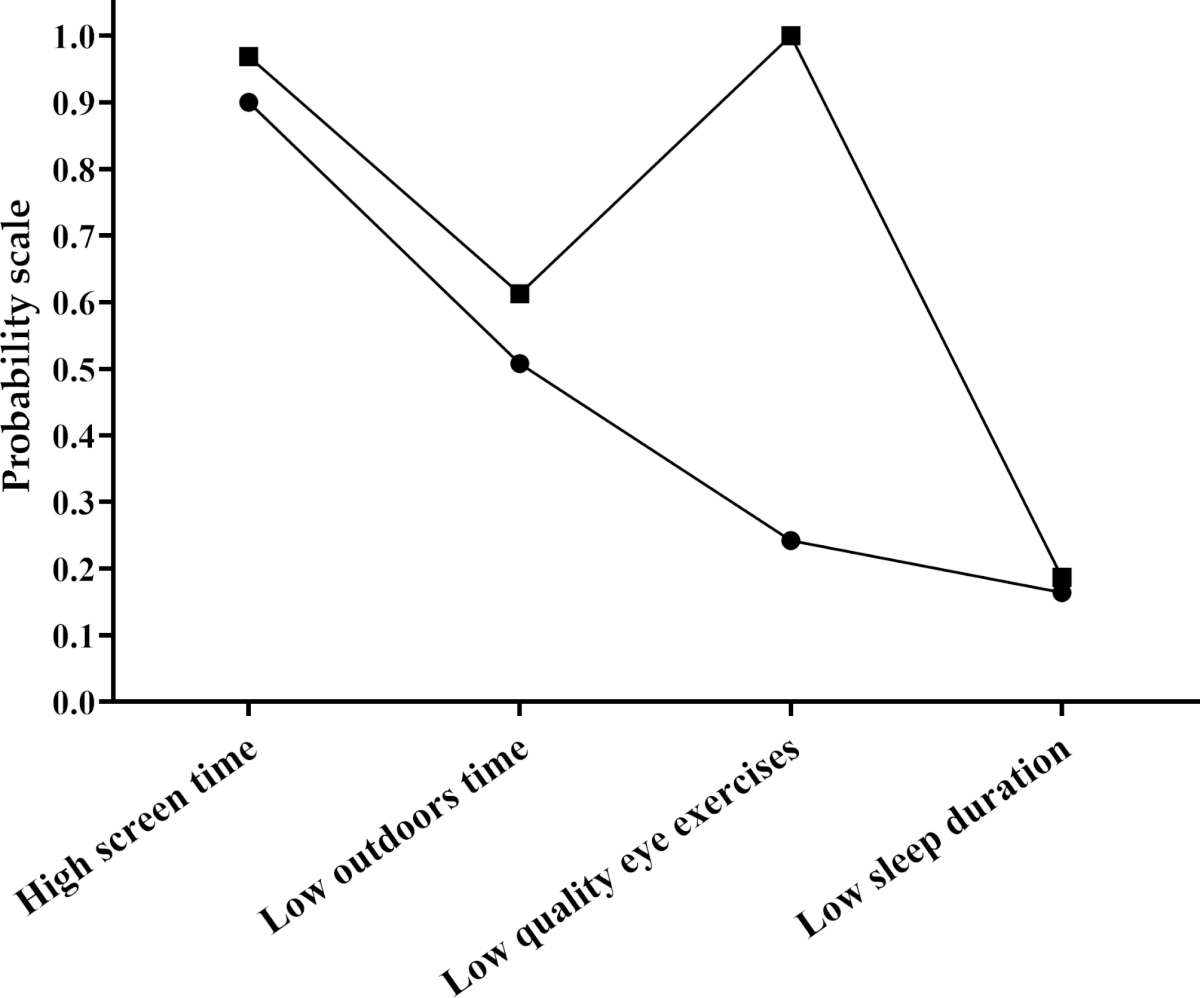
Two patterns of four behaviors of the best-fitting model. ■ high risk pattern (828, 41.1%); • low risk pattern (1186, 59.9%)

A total of 91.8% of the participants reported that they had spent 2 h or more per day on digital screen time and 52.3% reported an outdoor time of less than 2 h per day. In addition, 43.7% did reported that they had performed low-quality Chinese eye exercises in past years. Only 17.0% of the participants reported a sleep duration of shorter than 7 h per night. The estimated probabilities of the four behavioral risk factors among the two identified latent patterns are shown in Fig. 1. In this study, 41.1% of the overall study sample were assigned into the high-risk class, indicating that they were more likely to have risky behaviors of myopia. The high-risk class had a higher likelihood of having digital screen time for 2 h or more (96.9%), spending time outdoors less than 2 h per day (61.3%), sleeping less than 7 h per night (18.7%) and performing low-quality Chinese eye exercise (100.0%). On the other hand, 1186 (59.9%) participants were assigned into the low-risk group, which had lower probabilities of being affected by the four myopia risky behaviors.

The characteristics of demographic characteristics and ocular biometric parameters for the two behavioral patterns are demonstrated in Table 3. In brief, students in the high-risk class had longer ALs and higher AL/CR ratios compared with their counterparts in the low-risk class. There were no significant differences in CRs and ACDs between the two classes (both *P* > 0.05). Univariate logistic regression models revealed that the risk of myopia (*OR* = 2.19, 95% *CI* = 1.52–3.14), high myopia (*OR* = 1.43, 95% *CI* = 1.14–1.78) and AL/CR ratio of more than 3.0 (*OR* = 1.82, 95% *CI* = 1.22–2.72) were significantly higher in the high-risk class (all *P* < 0.05). These associations remained significant after adjusting for potential confounders such as sex, habitual residence, household structure and parental education levels in multivariate analysis (all *P* < 0.05, Table 4).

**Table 3:** Demographic and myopia biometry parameters for each of the two clusters

Variables	High-risk class(%)	Low-risk class(%)	χ^2^/t	*P*
Sex				
Male	261(41.0)	376(59.0)	0.01	0.93
Female	567(41.2)	810(58.8)		
Ethnicity				
Han	201(40.0)	302(60.0)	0.37	0.54
Minority	627(41.5)	884(58.5)		
Local resident				
Yes	647(42.0)	892(58.0)	2.32	0.13
No	181(38.1)	294(61.9)		
Habitual residence				
Rural	262(37.8)	431(62.2)	4.77	**0.03**
Urban	566(42.8)	755(57.2)		
Father’s education level				
< High school degree	655(42.6)	884(57.4)	5.65	**0.02**
≥ High school degree	173(36.4)	302(63.6)		
Mother’s education level				
< High school degree	713(42.6)	959(57.4)	9.54	**0.002**
≥ High school degree	115(33.6)	227(66.4)		
AL, mm^#^	24.89 ± 1.15	24.77 ± 1.24	2.19	**0.03**
CR, mm^#^	7.85 ± 0.36	7.88 ± 0.28	-1.75	0.08
AL/CR^#^	3.17 ± 0.14	3.15 ± 0.15	4.02	**< 0.001**
ACD, mm^#^	3.63 ± 0.24	3.61 ± 0.25	1.68	0.09

Bolded number represents the *P* < 0.05. ^#^ data was presented as mean ± standard deviation. AL, axial length; CR, corneal radius; ACD, anterior chamber depth

**Table 4:** The association of two clusters with myopia

Variables	*OR*(95% *CI*)	*P*	*OR(*95% *CI*)^*^	*P* ^*^	*OR(*95% *CI*)^#^	*P* ^*#*^
Myopia						
Low-risk class	1.00		1.00		1.00	
High-risk class	2.19(1.52–3.14)	**< 0.001**	2.20(1.53–3.17)	**< 0.001**	2.22(1.54–3.20)	**< 0.001**
High myopia						
Low-risk class	1.00		1.00		1.00	
High-risk class	1.43(1.14–1.78)	**0.002**	1.45(1.16–1.82)	**< 0.001**	1.46(1.16–1.83)	**0.001**
Higher AL/CR						
Low-risk class	1.00		1.00		1.00	
High-risk class	1.82(1.22–2.72)	**0.004**	1.84(1.23–2.76)	**0.003**	1.84(1.23–2.76)	**0.003**

Bolded number represents the *P* < 0.05. ^*^ Adjusted for habitual residence, household structure and parental education levels. ^#^ Adjusted for sex, habitual residence, household structure and parental education levels. *OR*: odds ratio; *CI*: confidence interval

## Discussion

The present study investigated the associations between four major behavioral risk factors and myopia and ocular biometric parameters in young adults from a university-based sample, using the LCA method. We found that Chinese university students showed differential risks of myopia and could be subdivided into high- and low-risk clusters based on four behavioral variables. The LCA model identified two patterns and significant differences were revealed in the prevalence of myopia, mean ALs and AL/CR ratios. These findings may help clinicians and public health practitioners understand the behavioral risk pattern of myopia from a person-centered perspective, thus strengthening the importance of timely intervention and reducing the incidence of myopia in school students.

Although several behavioral risk factors have been recognized to play a major role in myopia development, there is a lack of appropriate approaches which could integrate them in myopia risk assessments and subsequent assist early interventions. The LCA is initially an unsupervised machine learning approach which can be used to screen high-risk populations of a certain disease [24]. The LCA assumes that a heterogeneous population are composed of a mixture of aggregates and a latent class variable determines the optimal model. The LCA could achieve “dimensionality reduction” in the analysis of high dimensional data and cluster at the individual level with the help of goodness-of-fit evaluation metrics [12]. The latent classes extracted by the LCA reflect the comprehensive effects of influencing factors included in the analysis. As a multidisciplinary approach, LCA has its advantages for evaluating the risks of diseases affected by multiple risk factors, especially in psychology [25]. Although the LCA has been widely used in medical research, no attempts have been made in myopia research [26]. Our study filled the gap of knowledge by successfully clustering the subpopulations of students in terms of myopia risks and identifying the characteristics of each clustered population using the LCA method.

The overall prevalence of myopia in this study is 91.8%, which is much higher than the 80.5% myopia rate of high school students announced by the National Health and Construction Commission of China [27]. However, these prevalence rates could not be compared directly due to the differences in age range of the study population, sampling frame and measurement of myopia. Ocular biometric parameters such as AL and AL/CR ratios have been regarded as an endophenotype of refractive error [28, 29]. In our study, not only myopia but also ocular biometric parameters were treated as outcome measures. We found that higher prevalence of myopia together with longer ALs and higher AL/CR ratios were more common in high-risk class, confirming that these behavioral risk factors could influence myopia development to various extent [9, 30–32]. However, the behavioral risk factors were not consistently associated with myopia in different populations. For example, an analysis of six population-based studies suggested that digital screen time was not significantly related to ALs [5]. Besides, the longitudinal Raine Study explored the 12-year sleep trajectory of participants from childhood to adolescence and did not observe the association between sleep behavior and ALs [33]. These discrepancies in literatures exist probably because those previous results were mainly generated from variable-centered analysis. In variable-centered analysis, a single behavior only has minor effects on health outcomes and different study samples might have different susceptibilities to the same exposures, resulting in conflicting findings among various studies. The LCA is a person-centered analysis and might conquer the defects in variable-centered analysis. With the help of the LCA, our study successfully identified two behavior patterns and demonstrated that the risk of myopia significantly increases in high-risk behavioral class regardless of a single behavior. The findings might have implications in future study design and selection of participants who are at high risk of myopia in clinical research.

Although we had only included the students from a single university, the study sample can be representative of all university freshmen in this area as Dali University is the only university in Dali. In addition, Dali University is one of the few comprehensive universities in Yunnan and the selection of these students in this university would facilitate the comprehensive understanding of the eye health of college students in different majors. We had included more female than male students in this study and the study sample might not be representative of the Chinese college students in terms of sex ratio. However, the disparities in sex ratio would not significantly bias the findings as there was little evidence showing that sex could modify the associations between lifestyle-related variables such as time outdoors and myopia [34, 35].

The main strength of the study included its university-based sample and the use of LCA assessment model, which had good accuracy and could be applied to identify risk pattern of myopia. Nevertheless, we had to acknowledge several limitations of this study. First, it was a cross-sectional design and could not determine the causal relationship between exposures and outcome measures. Second, information regarding behavioral risk factors was collected using self-administered questionnaires and information biases such as recall biases or social desirability bias were inevitable. Third, only four behavioral risk factors were included in this study and other behavioral risk factors such as dietary factors may also contribute to myopia but were not captured in this study, resulting in a less accurate classification of the high- and low-risk classes. Last but not least, refractometry was performed without cycloplegia, which may over estimate myopic refractive error.

## Conclusion

In summary, this study successfully identified two patterns of myopia based on four major behavioral risk factors for myopia. The findings may help clinicians understand the risk of myopia among students with different behavioral patterns and facilitate the early identification of high-risk individuals, and encourage precise monitoring and management among high-risk population to alleviate the occurrence and progression of myopia among school students.

## Data Availability

The datasets generated and/or analysed during the current study are not publicly available, but are available from the corresponding author on reasonable request.
